# Correction: Green synthesis of AuNPs using *Cistanche tubulosa* extract and their broad-spectrum antimicrobial, antiparasitic, and scolicidal activities

**DOI:** 10.1039/d6ra90101h

**Published:** 2026-08-12

**Authors:** Hanieh Karimi, S. Ebrahim Seifati, Damoun Razmjoue, Soraya Ghayempour

**Affiliations:** a Department of Arid Land and Desert Management, School of Natural Resources and Desert Studies, Yazd University Yazd Iran seifati@yazd.ac.ir; b Phytochemistry Research Center, Shahid Beheshti University of Medical Sciences Tehran Iran; c Medicinal Plants Research Center, Yasuj University of Medical Sciences Yasuj Iran; d Department of Textile Engineering, Faculty of Engineering, Yazd University Yazd Iran

## Abstract

Correction for ‘Green synthesis of AuNPs using *Cistanche tubulosa* extract and their broad-spectrum antimicrobial, antiparasitic, and scolicidal activities’ by Hanieh Karimi *et al.*, *RSC Adv.*, 2025, **15**, 49924–49932, https://doi.org/10.1039/d5ra08159a.

The authors regret that one of the affiliations (affiliation *b*) was incorrectly shown in the original manuscript. The corrected list of affiliations is as shown herein.

The Royal Society of Chemistry apologises for these errors and any consequent inconvenience to authors and readers.

